# The effect of age on sagittal plane profile of the lumbar spine according to standing, supine, and various sitting positions

**DOI:** 10.1186/1749-799X-9-11

**Published:** 2014-02-27

**Authors:** Eui Seok Lee, Cheol Woong Ko, Seung Woo Suh, Suresh Kumar, Il Kuy Kang, Jae Hyuk Yang

**Affiliations:** 1 Department of Oral and Maxillofacial Surgery, Korea University, Guro Hospital, Guro 2-dong, Guro-gu, Seoul, 152-703, South Korea; 2 Advanced Biomedical and Welfare Technology R&BD Group, Korea Institute of Industrial Technology (KITECH), 89 Yangdaegiro-gil, Ipjang-myeon, Seobuk-gu, Cheonan-si, Chungcheongnam-do, 331-822, South Korea; 3 Scoliosis Research Institute, Department of Orthopaedics, Korea University, Guro Hospital, Guro 2-dong, Guro-gu, Seoul, 152-703, South Korea; 4 Department of Radiology, Korea University, Guro Hospital, Guro 2-dong, Guro-gu, Seoul, 152-703, South Korea

**Keywords:** Spine, Lumbar lordosis, Position change, Age

## Abstract

**Background:**

The sagittal alignment of the spine changes depending on body posture and degenerative changes. This study aimed to observe changes in sagittal alignment of the lumbar spine with different positions (standing, supine, and various sitting postures) and to verify the effect of aging on lumbar sagittal alignment.

**Methods:**

Whole-spine lateral radiographs were obtained for young volunteers (25.4 ± 2.3 years) and elderly volunteers (66.7 ± 1.7 years). Radiographs were obtained in standing, supine, and sitting (30°, 60°, and 90°) positions respectively. We compared the radiological changes in the lordotic and segmental angles in different body positions and at different ages. Upper and lower lumbar lordosis were defined according to differences in anatomical sagittal mobility and kinematic behavior.

**Results:**

Lumbar lordosis was greater in a standing position (52.79° and 53.90° in young and old groups, respectively) and tended to decrease as position changed from supine to sitting. Compared with the younger group, the older group showed significantly more lumbar lordosis in supine and 60° and 90° sitting positions (*P* = 0.043, 0.002, 0.011). Upper lumbar lordosis in the younger group changed dynamically in all changed positions compared with the old group (*P* = 0.019). Lower lumbar lordosis showed a decreasing pattern in both age groups, significantly changing as position changed from 30° to 60° (*P* = 0.007, 0.007).

**Conclusions:**

Lumbar lordosis decreases as position changes from standing to 90°sitting. The upper lumbar spine is more flexible in individuals in their twenties compared to those in their sixties. Changes in lumbar lordosis were concentrated in the lower lumbar region in the older group in sitting positions.

## Background

Studies of the sagittal alignment and profile of the lumbar spine had been thought to be important because stress concentration in unbalanced sagittal spine can lead to pain (functional pathology) and degeneration of disc and facet joints [1-3]. This sagittal alignment and profile of the lumbar spine can be affected by degeneration due to aging process, postural changes according to the shape of sitting chair, and surgical treatment such as instrumentation [1,4].

Because of the abovementioned reasons, many studies have investigated variations in the sagittal profile of the thoracolumbar or lumbar spine according to different body positions or aging process [5-8]. However, previous studies had some limitations as follows: Firstly, most studies involved sagittal alignment in sitting and/or supine position, or at a 90° sitting position. These studies generally did not include other sitting positions that occur in the routine normal daily activities (although a few did include various positions) and did not give enough information on adaptional change of lumbar profiles because it was not examined with sequential angles; secondly, they used only indirect assessment methods with skin-mounted inclinometers or flexure curvature techniques [9,10]. These methods cannot measure bony angles of lumbar spine; therefore, the change of lumbar profiles reported in previous papers is indirect information; thirdly, the effect of the aging process on the degeneration on the disc and vertebrae is an important factor to lumbar sagittal profiles, but previous study is not designed to comparative study form considering the analysis of aging effect on various positions.

Therefore, we planned to perform a study to assess the sagittal lumbar profiles in different postures adapted to daily life (standing, supine, and sitting (30°, 60°, and 90°)). Furthermore, we investigated effects of aging on the lumbar sagittal profile by comparing lumbar spine parameters between younger (second decade of life) and older (aged more than 65 years) volunteers.

## Methods

### Statement of ethical approval

We certify that this study involving human subjects is in accordance with the Helsinki declaration of 1975 as revised in 2000 and that it has been approved by the relevant institutional Ethical Committee. This study was performed under the approval of the institutional board review (IRB No. MD 10024). This was a prospective, non-randomized case–control study. Appropriate institutional review board approval was obtained before the study began. All subjects were fully informed about the methods, purposes, and risks involved in the study protocol and provided written statement of informed consent.

### Subjects

The sample comprised healthy Korean volunteers who had had no low back pain in the previous 6 months and no history of spinal fracture, infection, tumor, or metabolic bone disease. They were able to position themselves appropriately for radiography. Before the study, we evaluated the standing lumbar spine anteroposterior and lateral radiographs for detecting fractures and deformities that could affect sagittal alignment and for detecting abnormal sagittal balance. After that, the whole spine lateral radiography in standing position was taken for the sagittal balance evaluation. The optimal sagittal balance was defined as falling of the C7 plumb line within 3 cm of the posterior edge of the first sacrum [5,11]. Volunteers with abnormal findings in radiography and sagittal balance were excluded.

Subjects were divided into two age groups with ten subjects each: young (second decade of life) and older (more than 65 years of age). The younger group included men aged 25.4 ± 2.3 years (height 175.3 ± 3.5 cm, weight 75.1 ± 8.2 kg). The older group included men aged 66.7 ± 1.7 years (height 168.8 ± 5.3 cm, weight 73.6 ± 8.5 kg). The body mass index was calculated as 24.4 ± 2.6 and 25.8 ± 1.9 in younger and older age groups, respectively (Table 1).

**Table 1:** Descriptive data of the young and old age groups

**Factors**	**Young age group**	**Old age group**	***P *** **values**
Enrolled number	10	10	
Sex	Male: 10	Male:10	
Age (years)	25.4 ± 2.3	66.7 ± 1.7	<0.001^¶^
Height (cm)	175.3 ± 3.5	168.8 ± 5.3	0.021^¶^
Weight (kg)	75.1 ± 8.2	73.6 ± 8.5	0.967^¶^
Body mass index	24.4 ± 2.6	25.8 ± 1.9	0.121^¶^
Disc degeneration^*^ (average value)	L1/2: 0.0	L1/2: 0.8	
	L2/3: 0.1	L2/3: 1.6	
	L3/4: 0.2	L3/4: 1.8	
	L4/5: 0.2	L4/5: 1.7	
		L5/S1: 0.0		L5/S1: 1.8

For statistical analysis, Mann–Whitney test was used. ^*^For grading disc degeneration, simple radiography was used and the guideline by Lawrence JS et al. was used. Grade 1 is defined as the status of a slight anterior wear of the vertebral body and osteophyte formation; grade 2 is defined as the status of definite anterior wear of vertebral body and osteophyte formation; grade 3 is defined as the status of osteophyte formation and narrowing of disc; grade 4 is defined as the status of large osteophyte formation, marked disc narrowing, sclerosis of vertebral plates, and posterior subluxation.

For confirming the proper positioning and spine balance in the laboratory chair, whole spine lateral radiography was used in all subjects. For a clearer evaluation on the lumbar component compared to the cervical and thoracic spine, the X-ray beam for radiography was focused on the second lumbar vertebrae. Whole spine lateral radiographs were obtained for all subjects in different positions.

### Design of the laboratory chair device and examination conditions for radiography

**Figure 1:**
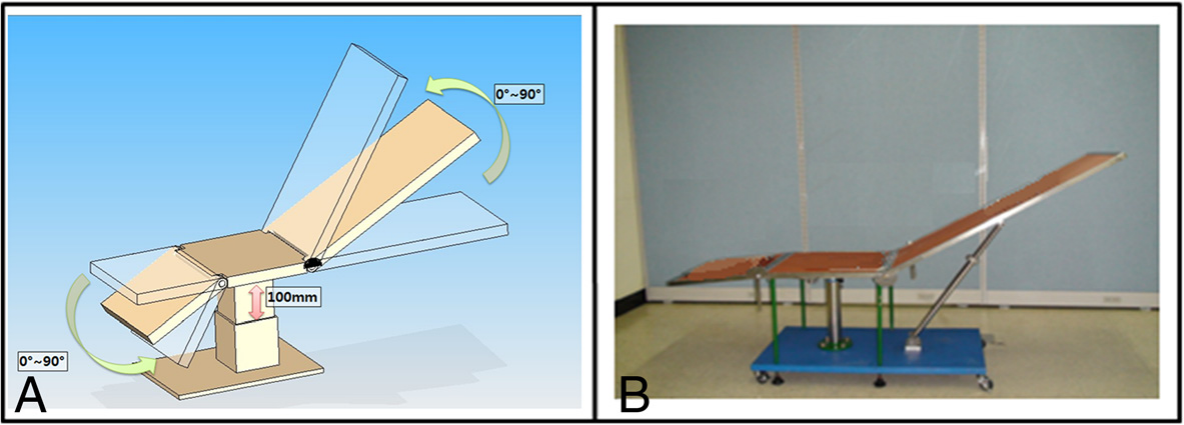
**A simplified chair device with three-segmental rigid plates for evaluating sagittal alignment with radiography. (A)** The chair was designed with a leg rest, a seat, and a seat back comprising three separate parts. The length, width, and thickness of the seat back, seat, and leg rest were 970 × 400 × 20, 500 × 400 × 20, and 450 × 400 × 20 mm, respectively. **(B)** Prototype of the chair device.

A special chair appropriate for a standard Korean figure was designed for this study (Report on the 5th human body measurement of Korean, Size Korea, 2004) [12] (Figure 1). The device comprised of three rigid plates, a seat back, a seat, and a leg rest. These three parts can be easily adjusted to different angles. A customized angle finder was attached to the chair for precise measurement of the angles [13]. The lumbar sagittal profile was assessed in different postures.

To ensure consistency in radiography techniques and decrease the effect of position, we adhered to the following standardized protocol. For the standing position, all subjects were instructed to stand with arms crossed while looking forward at a 15° angle upward direction. They were also asked to keep their hips and knees extended [14]. For the supine position, the hip and knee joints were extended and the arms were crossed. The subjects also crossed their arms for sitting positions. The subjects were asked to place their hips at the inner endpoint of the chair's seat plate to minimize changes in lumbar shape due to sitting habits. Moreover, we ensured that the upper back and head were in complete contact with the seat back of the chair. The subjects were given approximately 5 min to relax between positions. Spine radiographs were obtained for five postures: standing, supine, and sitting (30°, 60°, and 90°), respectively. We used DRS-8000 (Digital Radiography System, Listem Corp., Seoul, Korea) for radiography, and the image files were converted to the DICOM format for analysis. Analysis of radiography data.

**Figure 2:**
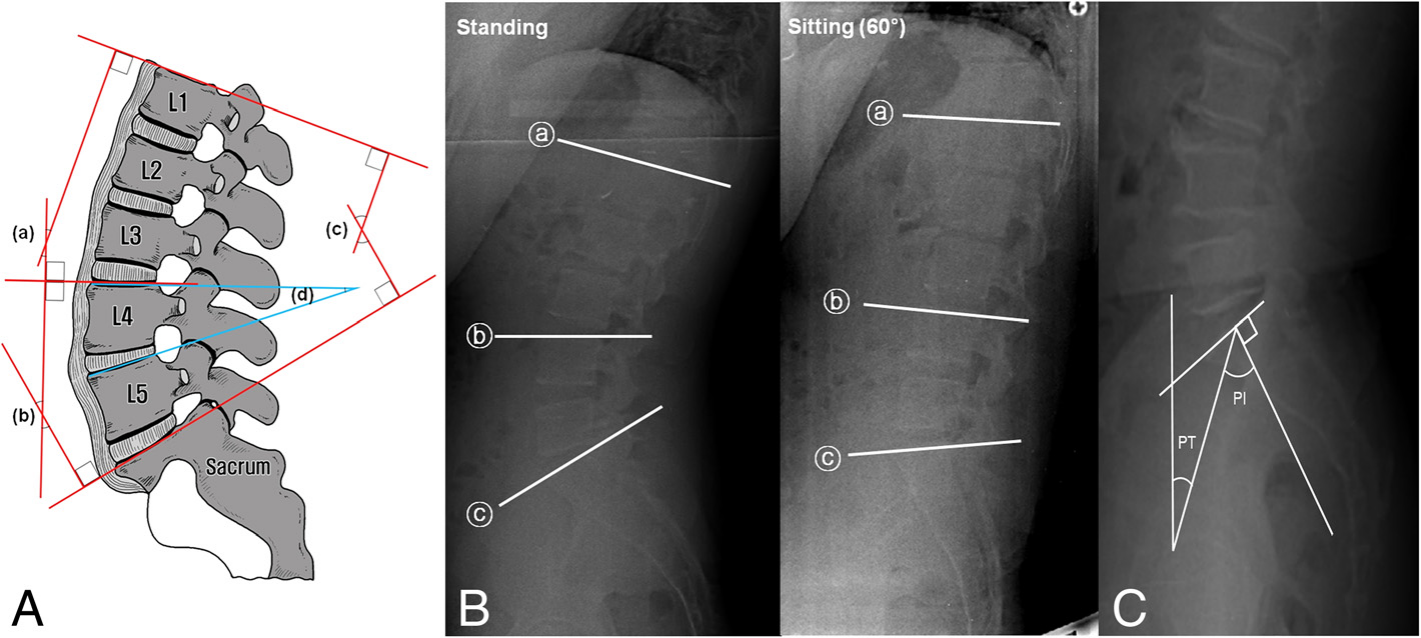
**Lumbar lordosis and segmental angle measurements, radiographies on end-plate angle measurements and pelvic tilt and pelvic incidence measurements. (A)** Measurement of lumbar lordosis, upper and lower lumbar lordosis, and segmental angles. (c) The lumbar lordotic angle is the angle between the upper plate of the first lumbar and first sacral vertebral bodies. (a) The upper lumbar lordosis is the angle between the upper plate of the first and fourth lumbar vertebral bodies. (b) The lower lumbar lordosis is the angle between the upper plate of the fourth lumbar and first sacral vertebral bodies. Each segmental angle was measured as the angle between the upper plates of two adjacent vertebral bodies. **(B)** Measurement references for end-plate angle based on radiography. In the standing position, lumbar lordosis (angle between ⓐ and ⓒ), upper lumbar lordosis (angle between ⓐ and ⓑ), and lower lumbar lordosis (angle between ⓑ and ⓒ) were 59.09°, 17.46°, and 44.82°, respectively. After position was changed to sitting with the seat back at 60°, lumbar lordosis, upper lumbar lordosis, and lower lumbar lordosis changed to 8.26°, -5.15°, and 15.03°, respectively. **(C)** Radiography showing the measurement of pelvic tilt (PT) and pelvic incidence (PI). PT is defined as the angle between the line joining the hip axis and the midpoint of the S1 end plate and the reference vertical line. PI is defined as the angle between the line joining the hip axis and the midpoint of the S1 endplate and the line orthogonal to the S1 end plate.

This study concentrated on the influence of sagittal profile on the lumbar spine. We analyzed lumbar spine radiographs using a picture archiving and communication system (PACS, LG Infinity, Korea). Lumbar lordotic angles (*φ*) and segmental angles in the sagittal plane were measured (Figure 2) [6,7,15,16].

To identify the effects of angles on lumbar sagittal profile, lumbar lordosis was sub-classified as upper lumbar lordosis (ULL, the angle between the upper end plate of the first lumbar vertebrae and the upper end plate of the fourth lumbar vertebrae) and lower lumbar lordosis (LLL, the angle between the upper end plate of the fourth lumbar vertebrae and the upper end plate of the first sacral vertebrae) by considering segmental angular motion, anatomical features such as facet joint orientation, and differences in kinematic behavior of the lumbar spine [15,17-19]. The angles were measured three times each by two orthopedic spine specialists, and the average values were used for final analysis.

Disc degeneration was evaluated with the grading system by Lawrence JS [20]. And pelvic incidence and tilt according to posture were also evaluated (Figure 2) [21].

### Statistical analysis

Statistical analysis was performed using SPSS software (version 17.0; SPSS; IL, USA). The average values were compared and analyzed using the non-parametric method such as the Mann–Whitney *U* test, Wilcoxon signed rank test, and Kruskal-Wallis test. *P* values less than 0.05 were considered statistically significant. Because the sample was small, statistically significant data were reanalyzed with the GPower program (version 3.1) [22]. Statistical data are presented with *P* values (power of the statistical result). Intra-observer reliability and inter-observer reliability were tested with intraclass coefficients (ICCs) computed using a two-way mixed model and absolute agreement. Strong reliability was defined as an ICC in the range of 0.8–1.00 [23].

## Results

Mean values of intra-observer reliability and inter-observer reliability were 0.968 and 0.892, respectively, and all ICCs were greater than 0.8 (Table 2). Lumbar lordosis values and segmental angles for both age groups are summarized in Table 2.

**Table 2:** Results of intra- and inter-observer reliability test

**Posture**	Intra-observer reliability^*^	Inter-observer reliability^*^
Standing	0.97	0.89
Sitting		
30°	0.98	0.91
60°	0.96	0.88
90°	0.96	0.88
Supine	0.97	0.90

^*^The intra- and inter-observer reliability was tested using the intraclass coefficient (ICC) test with a set-up of a two-way-mixed and absolute agreement. The values of the ICC test more than 0.8 could be thought to be strongly reliable.

**Figure 3:**
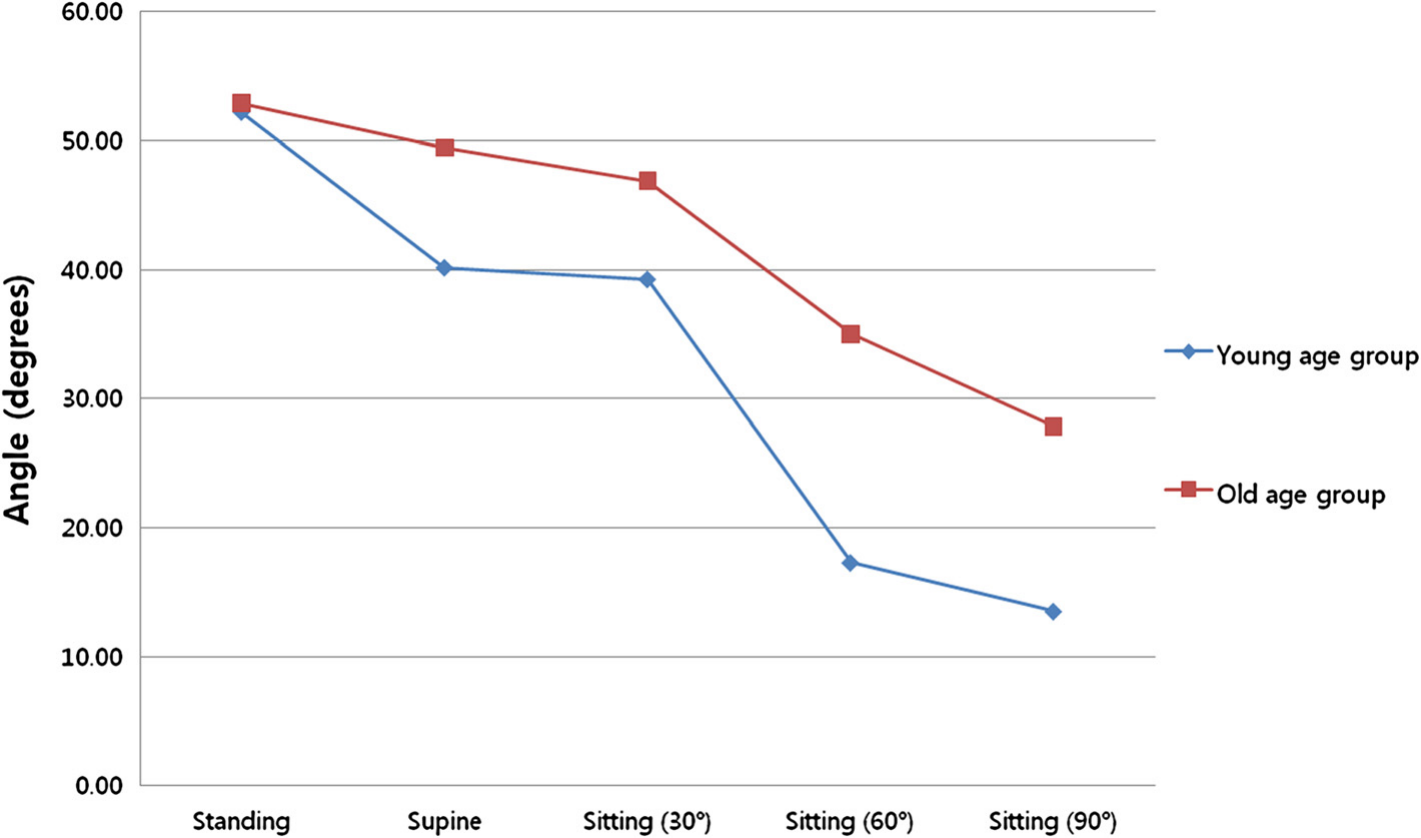
**Changes in lumbar lordosis according to posture in the two age groups.** Lumbar lordosis decreased as posture changed from standing to supine, and as sitting angle increased (from 30° to 90°) in the young and older groups. In the young group, lumbar lordotic angles were relatively greater when posture changed from standing to supine and from sitting at 30° to sitting at 60°.

Radiographs of the young group revealed that a lumbar lordotic angle (*φ*) of 52.79° ± 7.95° was the largest in the standing position. Lumbar lordosis tended to decrease as position changed from standing to supine and as sitting angle increased (from 30° to 90°, Figure 3). When the position changed from standing to supine and from sitting at 30° to sitting at 60°, lumbar lordosis showed a greater change in angles compared to other postural changes, by 12.04° ± 11.37° and 21.95° ± 8.03° (Table 3).

**Table 3:** Summary of lumbar lordotic angle and end-plate angles of lumbar spine based on whole spine lateral radiography

**Group**	**Factors**	**Standing**	**Supine**	**Sitting**		
				**30°**	**60°**	**90°**
Young age group	ф 1	3.81 ± 2.94	-0.27 ± 1.93	-1.78 ± 3.85	-2.33 ± 4.13	0.47 ± 3.70
	ф 2	5.53 ± 2.86	1.98 ± 2.95	1.40 ± 4..20	1.04 ± 2.77	0.84 ± 3.74
	ф 3	9.32 ± 1.78	6.30 ± 3.78	5.56 ± 2.46	2.73 ± 2.34	2.44 ± 3.78
	ф 4	12.51 ± 4.00	12.64 ± 4.52	12.83 ± 4.15	4.54 ± 2.56	1.87 ± 3.84
	ф 5	22.38 ± 6.12	20.50 ± 3.09	22.14 ± 5.66	12.45 ± 3.96	8.96 ± 4.47
	Ф	52.20 ± 7.95	40.16 ± 8.84	39.25 ± 6.92	17.30 ± 7.10	13.52 ± 11.61
	ULL	18.66 ± 4.32	8.01 ± 7.20	5.18 ± 7.24	1.44 ± 6.64	3.74 ± 9.35
	LLL	34.88 ± 7.40	33.14 ± 5.45	37.97 ± 7.29	16.99 ± 4.43	10.56 ± 6.35
Old age group	ф 1	1.78 ± 3.77	0.12 ± 2.97	-0.18 ± 4.02	0.28 ± 4.32	-0.11 ± 3.60
	ф 2	5.22 ± 4.99	2.78 ± 3.51	4.10 ± 4.34	3.14 ± 4.61	2.54 ± 4.13
	ф 3	7.51 ± 4.19	7.39 ± 3.80	8.41 ± 4.32	6.38 ± 4.88	4.25 ± 4.22
	ф 4	14.68 ± 3.23	14.88 ± 3.86	11.97 ± 4.85	8.75 ± 3.07	6.83 ± 5.18
	ф 5	24.98 ± 8.37	23.42 ± 6.77	22.98 ± 7.64	16.75 ± 7.39	14.61 ± 4.06
	Ф	53.90 ± 15.90	49.50 ± 13.12	46.91 ± 12.63	35.02 ± 10.26	27.87 ± 9.15
	ULL	14.51 ± 9.72	10.28 ± 7.83	12.33 ± 10.23	9.80 ± 10.66	6.68 ± 9.66
	LLL	39.66 ± 8.75	38.30 ± 8.44	34.94 ± 10.41	25.50 ± 8.29	14.61 ± 4.06

The values are in degrees, and the mark ‘°’ means the degree; ф1 means the first segmental angle which was measured between upper end plate of the first lumbar and upper end plate of the second lumbar vertebrae; ф2 means the second segmental angle which was measured between the upper end plate of the second lumbar and upper end plate of the third lumbar vertebrae; ф3 means the third segmental angle which was measured between the upper end plate of the third lumbar and upper end plate of the fourth lumbar vertebrae. ф4 means the fourth segmental angle which was measured between the upper end plate of the fourth lumbar and upper end plate of the fifth lumbar vertebrae; ф5 means the fifth segmental angle which was measured between the upper end plate of the fourth lumbar and upper end plate of the first sacral vertebrae. Ф means lumbar lordosis which was measured between the upper end plate of the first lumbar and upper end plate of the first sacral vertebrae. ULL and LLL means upper lumbar lordosis and lower lumbar lordosis.

**Figure 4:**
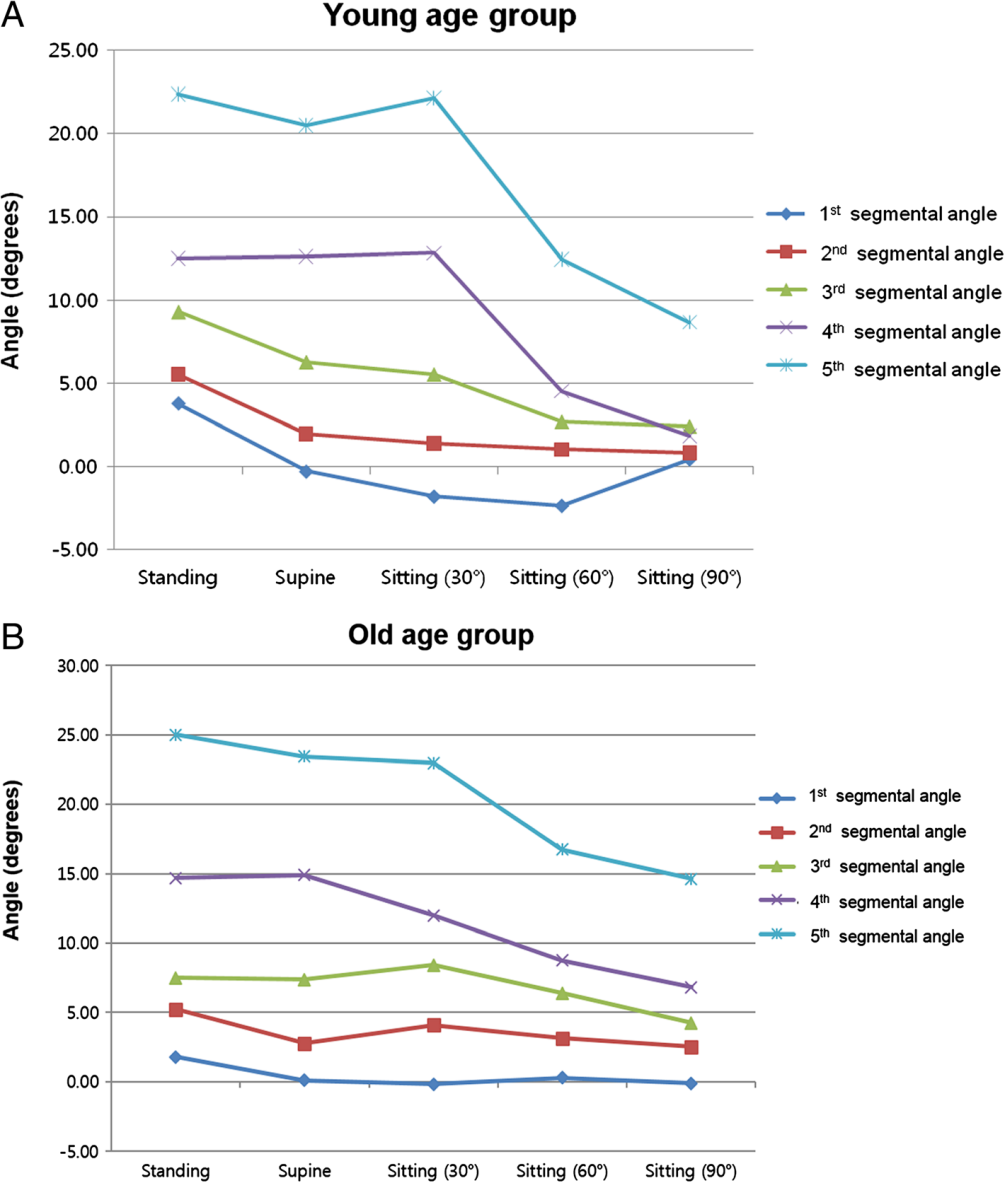
**Changes in segmental angles according to posture in two age groups. (A)** Relatively larger changes in the first, second, and third segmental angles were observed when the posture of younger subjects changed from standing to supine. In contrast, relatively smaller changes were observed in the fourth and fifth segmental angles. Relatively larger changes were observed in the fourth and fifth segmental angles when sitting posture changed from 30° to 60°. **(B)** Insignificant changes in the first, second, and third segmental angles were detected in the older subjects. In contrast, various changes were observed in the fourth and fifth segmental angles according to postural changes.

**Figure 5:**
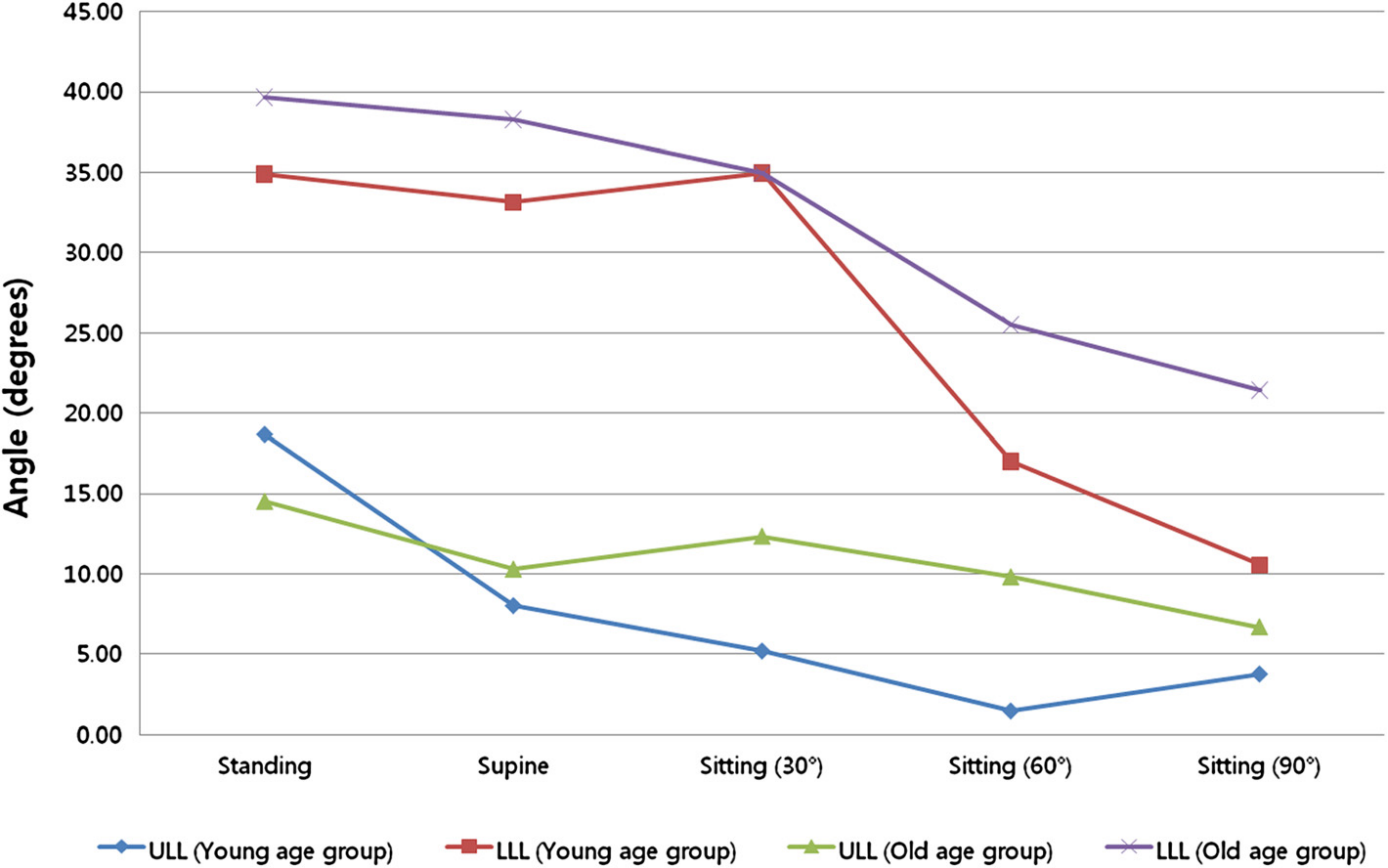
**Changes in upper and lower lumbar lordosis according to posture in two age groups.** Lordosis was measured in upper and lower regions of the spine, defined according to anatomical characteristics. Kyphotic and lordotic changes in upper lumbar lordosis varied according to postural changes in younger subjects. However, nonsignificant changes were observed in older subjects. No changes in lower lumbar lordosis were observed during change from a standing to supine position in either age group. In contrast, a drastic change was identified in sitting positions in the young group compared to the older group.

The first, second, and third segmental angles of ULL tended to decrease as posture changed from standing to supine, with statistically significant differences (*P* = 0.001 (0.99), 0.003 (0.96), and 0.011 (0.83), respectively) (Figure 4A). However, the fourth and fifth segmental angles of LLL exhibited no statistically significant differences as posture changed from standing to supine (*P* = 1.00 and 0.557, respectively). In contrast, these segmental angles decreased significantly during changes in sitting posture, from 30° to 60° and from 60° to 90° (*P* = 0.001 (0.99), 0.011 (0.72) for the fourth segmental angle; *P* = 0.006 (0.99), 0.001 (0.80) for the fifth segmental angle) (Figure 4A). These outcomes were similar to the changes observed in ULL and LLL (Figure 5).

In the older group, a lumbar lordotic angle of 53.90° ± 15.90° was the largest in the standing position. Lumbar lordosis gradually decreased as position changed from standing to supine, and as sitting angle increased (from 30° to 90°) (Figure 3). In the younger group, differences in the lumbar lordotic angle in supine and 60° sitting and 90° sitting positions were statistically significant (*P* = 0.043 (0.54), 0.002 (0.99), 0.011 (0.89)) (Table 4).

**Table 4:** Results of statistical analysis between younger and older groups

**Measurement factors**	**Young age group**	**Old age group**	***P *** value* (power of statistical results)†
1. Standing Ф	52.20 ± 7.95	53.90 ± 15.90	0.631
2. Supine Ф	40.16 ± 8.84	49.50 ± 13.12	0.043 (0.54)
3. Sitting Ф (at 30°)	39.25 ± 6.92	46.91 ± 12.63	0.143
4. Sitting Ф (at 60°)	17.30 ± 7.10	35.02 ± 10.26	0.002 (0.99)
5. Sitting Ф (at 90°)	13.52 ± 11.61	27.87 ± 9.15	0.011 (0.89)

*For comparing the mean values between groups, the Mann–Whitney *U* test was used. The *P* values under 0.05 was considered to be significant. †The evaluation of power on statistical results. GPower program set up with alpha values as 0.05 was used. The values are in degrees and the mark ‘°’ means the degree. Ф means the lumbar lordotic angle which was measured between the upper end plate of the first lumbar and upper end plate of first sacrum. ULL and LLL mean upper lumbar lordosis and lower lumbar lordosis, respectively.

The first, second, and third segmental angles of ULL were almost the same, exhibiting no statistically significant differences regardless of posture (*P* = 0.781, 0.551, 0.152) (Figure 4B). In contrast, the fourth and fifth segmental angles of LLL tended to decrease as posture changed. Although the fourth segmental angle showed no significant difference in standing and supine postures (*P* = 0.625), a statistically significant difference was detected between supine and 30° sitting postures (*P* = 0.015 (0.59)). The fourth segmental angle gradually decreased in all sitting positions, in a linear pattern (Figure 4B). The fifth segmental angle showed an insignificant difference as position changed from standing to supine and from supine to 30° sitting (*P* = 0.375, 0.557). However, it showed a statistically significant decrease as sitting position changed from 30° to 60° and from 60° to 90° (*P* = 0.001 (0.77), 0.035 (0.40)). These outcomes are similar to the changes observed in ULL and LLL (Figure 5).

Pelvic incidence was measured as 45.0° ± 7.1° and 50.1° ± 9.3° in young and old age groups. Pelvic tilt was measured as 13.3° ± 7.3°, 8.0° ± 2.2°, 18.6° ± 7.5°, 35.6° ± 7.4°, and 40.3° ± 10.9° in supine, standing, and sitting at 30°, 60°, and 90° in young age groups, respectively. In the old age group, pelvic tilt was measured as 20.3° ± 6.7°, 11.4° ± 7.2°, 21.1° ± 8.0°, 30.0° ± 8.9°, and 37.3° ± 9.1° in supine, standing, and sitting at 30°, 60°, and 90°, respectively.

The disc degeneration was more progressed in the old age group and the average grade of disc degeneration was described in Table 1.

## Discussion

In view of the sagittal balance, the lumbar spine is considered the major site because it has a larger mobile segment in the sagittal plane because of the anatomical morphology of its facet joints [24-26]. For these reasons, sagittal balance of the spine has been extensively studied with regard to the lumbar spine. Adams [1] suggests that there is a possible situation of ‘functional pathology’ which means developing of pain by stress concentration without mechanical or morphological deterioration in the spine. Likewise, abnormal flexion or extension by hypholordosis or hyperlordorsis can lead to more stress on facet joint and lead to early degeneration of spine in biomechanical study [27,28]. In anatomical study, by Umehara et al. [4], they suggesting that the hypholordosis caused by posterior instrumentation lead to stress on adjacent spinal segments and it can act as cause of post-operative pain.

The importance of sagittal balance and motion of lumbar spine has caught the eye of many authors who performed various studies on this aspect, by categorizing the lumbar spine into the upper and lower lumbar, and superior and inferior lumbar spine [9,29]. Additionally, some authors have performed studies on this aspect of lumbar spine in various postures that include standing, sitting, and supine because younger individuals nowadays spend more time sitting in various postures while at work [6-8]. Dolan et al. [30] performed a study on commonly adopted postures and their effects on the lumbar spine. In their study, he observed the lumbar motion range using skin-inclinometer from standing to various sitting postures and suggested that lumbar lordosis is decreased in sitting position. But this previous study had limitations; there was no direct radiological observation of the lumbar spine motion, and the evaluation of the lumbar spine motion was done indirectly using an electronic device without any observation regarding the facet joint motion. Adams et al. [31] also measured lumbar sagittal profile in forward and backward bending in a seated posture, but he also used the skin-inclinometer without confirming the spine radiologically. After these studies, various authors examined lumbar spine motion with radiography [6-8]. But their studies have not focused on the changes in the lumbar sagittal profile in various angled sitting positions. Their studies confirm the changes in the lumbar sagittal profile only in standing, supine, or sitting at 90°.

Therefore, the background data available for the present study were limited to investigations of subjects with back pain in various sitting postures.

We designed this study to verify the effects of standing, supine, and various sitting positions on the lumbar spine sagittal profile. We also intended to examine the effects of aging on the lumbar sagittal profile in different postures using a prospective, comparative approach.

In this study, lumbar lordotic angle was greatest in a standing position in both age groups, and there was no significant difference in lumbar lordosis between the two groups (*P* = 0.631). This result is similar to the results of studies on lumbar sagittal balance [9,10]. On the basis of this result, we infer that global lumbar profile was not affected by aging of discs, facet joints, or vertebral bodies [5]. However, when we separately analyzed lumbar lordotic angle as ULL and LLL, the distribution of ULL and LLL was different. The ratio of ULL to lumbar lordosis was significantly greater in the younger group (36.1% ± 7.3%) than in the older group (25.4% ± 12.9%) (*P* = 0.043 (0.70)). This finding suggests that the lumbar sagittal profile in a standing position is not affected by age, but components of the lumbar spine with distinct anatomical and biomechanical functions do appear to be affected by aging. Our findings are similar to previous studies' results. Burton [9] suggested that biomechanical consideration dictated the division of the lumbar spine into the upper region between the T12 and L4 vertebrae and a lower region between the L4 and S2 segments in his study of regional lumbar sagittal mobility. Rodriguez-soto et al. [29] also suggested that the superior and inferior lumbar level showed different kinematic behaviors.

We also tested this concept in changing positions from standing to supine and found that lumbar lordosis decreased regardless of age. However, the change was statistically significant in the younger group (*P* = 0.01 (0.99)) but not in the older group (*P* = 0.232). This can be explained by decreased spinal mobility due to aging. Many authors in their various biomechanical studies also suggested that the sagittal spine mobility decreased with age [9,10].

During position change from standing to supine, ULL changed by 10.65° ± 4.91° and 4.23° ± 5.21° in the younger and older groups, respectively; this was a statistically significant difference for both groups (*P* = 0.019 (0.84)). In contrast, LLL changed by 1.74° ± 9.60° in the younger group and 1.36° ± 8.09° in the older group, and there was no significant difference between the two groups (*P* = 0.912) (Figure 3B). To summarize, ULL appears to have had a greater influence than LLL on changes in lumbar lordosis during change from a standing to supine position in the younger group. ULL compensation is assumed to be less in the older group than in the younger group (Figure 5).

The decrease in lumbar lordotic angle and the difference between groups in the degree of change in ULL and LLL were also detected during the change from a standing to a 90°-sitting position (Table 4). ULL had a U-shaped pattern with significant variance during change from a standing to a 90° sitting position, but in the older subjects, ULL was flat (Figure 5). In both age groups, LLL tended to decrease. Continuous decrease in LLL was observed in the older group. However, LLL in the younger group did not decrease during change from supine position to 30° sitting position and it decreased within a wide range of values when subjects changed from a 30° sitting position to a 90° sitting position (Figure 5). Thus, ULL seems to act as an initial and general compensator of lumbar sagittal balance during changes in position, and LLL seems to have a significant role during sitting, especially at 60° and 90° angles. It was assumed that this control mechanism was affected by aging, for example because of the decrease in spinal mobility that is associated with old age [32,33]. As supporting clue for this suggestion, the degeneration of disc was higher in the older age group in the lower lumbar (Table 1).

To substantiate this premise and to further analyze the change in lordotic angle during sitting, we analyzed ULL and LLL with segmental angles. In the older group, we found no significant changes in the first, second, and third segmental angles for any other position (*P* = 0.781, 0.551, 0.152) (Figure 4B). In contrast, significant change was observed in the younger group (*P* = 0.001 (0.99), 0.003 (0.96), and 0.011 (0.83)). The first segmental angle in the younger subjects showed kyphosis during sitting at a 30° angle, but it gradually changed to lordosis during sitting at a 90° angle. The second segmental angle showed non-significant changes, and the third segmental angle tended to decrease. These findings indicate that the upper lumbar component was more flexible in the younger group than in the older group. As components of LLL, the fourth and fifth segmental angles constantly decreased in both age groups during change of position and we found a statistically significant decrease during change in sitting positions from 30° to 60° in both age groups (*P* = 0.007 (0.99) for the younger group and *P* = 0.007 (0.93) for the older group) and from 60° to 90° in the younger group (*P* = 0.009 (0.15)). The greatest change in lumbar lordotic angle occurred during change in sitting position from 30° to 60°, in both age groups (Figures 3 and 4). Considering that there was no statistically significant change in the upper lumbar segmental angle, the lower lumbar segmental angles can be thought of as a main compensator of the lumbar profile of older subjects during sitting.

This study had the following limitations: Firstly, the study sample was small. However, we endeavored to minimize distortion and generalization of the data by using various statistical tests and power analysis. Secondly, we only evaluated the male volunteers due to hazard of radiation during radiography evaluation. Although there is anatomical difference between male and female in spino-pelvic alignment and lumbar sagittal profile, we unified the gender of volunteer to obtain meaningful data in this study because the number of candidates for this study was limited and it was strictly controlled under IRB. Considering that this is first trial study on radiographic evaluation on lumbar sagittal profile in various postures, more study with large volume of candidates is necessary.

## Conclusions

Lumbar lordosis decreased with change in position from standing to supine and change in different sitting positions (seat back inclined at 30°, 60°, or 90°), regardless of age. However, there was a difference between younger and older subjects. This difference may be due to the disparity in flexibility or function of body parts. The upper lumbar spine is more flexible in individuals in their twenties compared to those in their sixties. Changes in lumbar lordosis during postural change are predicted to be evenly distributed in the upper and lower regions of the lumbar spine. In contrast, changes in lumbar lordosis were more concentrated in the lower lumbar region in the older group, especially when sitting. These anthropometric characteristics of the lumbar spine in elderly individuals and the response of the lumbar spine to different postures will provide insights for improving the design of chairs and for determining appropriate lumbar lordosis during spinal fusion surgery.

## Competing interests

We certify that there is no competing of interests with any financial organization regarding the material discussed in the manuscript. All authors in this study declare that they have no competing interests.

## Authors' contributions

ESL carried out the arrangement of the enrolled patients and drafted the manuscript as assistant of JHY. CWK and ESL participated in the design of the study and performed the statistical analysis as assistant of JHY. SWS carried out the arrangement of the enrolled patients and drafted the manuscript as the assistant of JHY. AWB participated in the design of the study. IKK participated in the design of the study. JHY participated in the design of the study, statistical analysis, and coordination and draft the manuscript as main author. All authors read and approved the final manuscript.
